# Editorial: Methods and applications in clinical and translational physiology

**DOI:** 10.3389/fphys.2023.1169544

**Published:** 2023-03-15

**Authors:** Gaetano Santulli, Christina M. Pabelick, Yih-Kuen Jan, Pierre Denise, Claudio de Lucia

**Affiliations:** ^1^ Department of Medicine, Wilf Family Cardiovascular Research Institute, Fleischer Institute for Diabetes and Metabolism (FIDAM), Albert Einstein College of Medicine, New York, NY, United States; ^2^ Department of Molecular Pharmacology, Einstein-Mount Sinai Diabetes Research Center (ES-DRC), Einstein Institute for Aging Research, Institute for Neuroimmunology and Inflammation (INI), Albert Einstein College of Medicine, New York, NY, United States; ^3^ Mayo Clinic, Rochester, MN, United States; ^4^ University of Illinois at Urbana-Champaign, Champaign, IL, United States; ^5^ Université de Caen Normandie, Inserm, COMETE U1075, CYCERON, CHU de Caen, Normandie University, Caen, France; ^6^ Geriatric Center Frullone, ASL (Azienda Sanitaria Locale-Local Health Authority) Napoli 1 Centro, Naples, Italy

**Keywords:** translational science, physiology, technologies, bioengineering, nanotechnology, biomedicine, advanced biomedical applications, applied physiology

The present Research Topic, entitled “Methods and Applications in Clinical and Translational Physiology” is part of the *Methods and Applications in Physiology* series. This series essentially aims at emphasizing the latest experimental techniques developed to understand the main fundamental questions in physiology. Therefore, this Research Topic covers the latest advances in technologies that will foster and advance scientific discoveries.

Our Research Topic has collected eight original articles and one review. The opening article is a randomized crossover study designed to determine the effect of hypoxia on metabolism in men with excess weight (Mekjavic et al.). Subjects participating in this study, conducted in Slovenia, undertook two trials, during which they were confined to normoxic vs*.* hypoxic conditions; the Authors concluded that the greater postprandial blood-glucose response following hypoxic confinement suggests the potential development of insulin resistance (Mekjavic et al.).

Then, two articles describe a novel device to measure pain (Boing-Messing et al.) and a new low-cost force plate to measure balance and postural sway (Lo et al.). The first device, called eEGG, which stands for electronic Egg, has been tested by German scientists and has been shown to have a strong correlation with the hand dynamometer, strongly suggesting that this new device could be used in medical practice (Boing-Messing et al.). Similarly, the force plate, evaluated both in young and elderly subjects was reliable when assessing several parameters related to the center-of-pressure (Lo et al.).

Next, we have a proteomic (Deng et al.) and a metabolomic (Sempore et al.) study. In the first one, aiming at identifying serum biomarkers clinically useful in acute aortic dissection, Deng, Liu, and others combine two established methodologies, namely, “isobaric Tags for Relative and Absolute Quantitation” (*iTRAQ*) and label-free methods, demonstrating that FGL1, MMP9, PI16, and Lumican may serve as potential biomarkers in patients with acute aortic dissection (Deng et al.). In the metabolomic study, Semporé and collaborators provide new insights into the biological modifications that accompany exercise in lower extremity artery disease, thereby contributing to a better understanding of the pathophysiology underlying walking impairment (Sempore et al.).

In the following study, Mexican researchers demonstrate that females undergoing peritoneal dialysis present higher serum levels of icodextrin metabolites–a specific fraction of dextrin (a starch-derived water-soluble glucose polymer) that has been successfully used as colloid osmotic agent–which may exert an increased colloid-osmotic pressure followed by reduced ultrafiltration volumes and increased blood volume and blood pressure (Paniagua et al.).

Then, we have two studies important in ophthalmology, mathematically proving the potential for mechanical damage to the corneal wound in cataract surgery (Qi et al.) and showing that, compared to gas tamponade, intravitreal silicone oil results in a significantly greater decrease in the thickness of the peripapillary retinal nerve fiber layer in patients with proliferative diabetic retinopathy (Wang et al.).

A systematic review examining the effects of duloxetine in patients with knee osteoarthritis concludes this Research Topic, showing that this treatment may be an effective treatment for improving pain and depressive symptoms in these patients (Zhou et al.).

